# Palladin is a novel microtubule-associated protein responsible for spindle orientation

**DOI:** 10.1038/s41598-017-12051-w

**Published:** 2017-09-18

**Authors:** Xiang Zhang, Xinlei Chen, Jing Liu, Xin Xu, Yuanliang Zhang, Zheng Ruan, Yinyin Xie, Qiuhua Huang, Tong Yin, Zhu Chen, Saijuan Chen

**Affiliations:** 1 State Key Laboratory of Medical Genomics, Shanghai Institute of Hematology, Rui Jin Hospital, Shanghai Jiao Tong University School of Medicine, 200025 Shanghai, China; 2 The National Research Center for Translational Medicine, Shanghai Jiao Tong University School of Medicine, 200025 Shanghai, China

## Abstract

Mitotic spindles, which consist of microtubules (MTs) and associated proteins, play critical roles in controlling cell division and maintaining tissue homeostasis. The orientation of the mitotic spindle is closely related with the duration of mitosis. However, the molecular mechanism in regulating the orientation of the mitotic spindles is largely undefined. In this study, we found that Palladin is a novel MT-associated protein and regulator of spindle orientation, which maintains proper spindle orientation by stabilizing astral MTs. *Palladin* depletion distorted spindle orientation, prolonged the metaphase, and impaired proliferation of HeLa cells. Results showed that *Palladin* depletion-induced spindle misorientation and astral MT instability could be rescued by constitutively active AKT1 or dominant negative GSK3β. Our findings revealed that Palladin regulates spindle orientation and mitotic progression mainly through the AKT1–GSK3β pathway.

## Introduction

Spindle orientation determines the axis of cell division. Thus, precise orientation and architectural integrity of the mitotic spindle is critical for mitosis^1^. Perturbation of mitosis due to spindle misorientation causes chromosome instability, which is an important aspect of tumorigenesis^2^. Proper spindle orientation depends mainly on astral microtubule (MT) stabilization, proper distribution of cell cortical complexes, and intact F-actin^3,4^. Various force generators and cytoskeleton-related kinases have been found to be involved in the regulation of spindle orientation^2,5–12^, but the molecular mechanism is largely unknown.

*Palladin* (*PALLD, RIG-K*) was originally cloned from the acute promyelocytic leukemia NB4 cell line induced by all-trans retinoic acid (ATRA) in our lab and was one of the up-regulated ATRA-inducing genes^13^. It has been characterized as a key actin-binding and microfilament-associated protein^14^. Furthermore, PALLD is responsible for cell morphology, mobilization, adhesion, invasion and metastasis of cancer cells^15–18^. However, the function of PALLD in regulating mitosis remains unknown.

In this study, we investigated the biological function of PALLD in spindle orientation and cell division. Our results showed that PALLD influenced cell proliferation mainly through regulating mitotic progression, especially at the metaphase. Furthermore, we identified that PALLD was a novel MT-associated protein involved in spindle orientation regulation. We also found that PALLD interacted with AKT1 via the third IgC domain to maintain AKT1-GSK3β activation and spindle orientation. These findings suggested that PALLD played important roles in spindle orientation maintenance and mitotic progression.

## Results

### PALLD regulates mitotic progression

**Figure 1:**
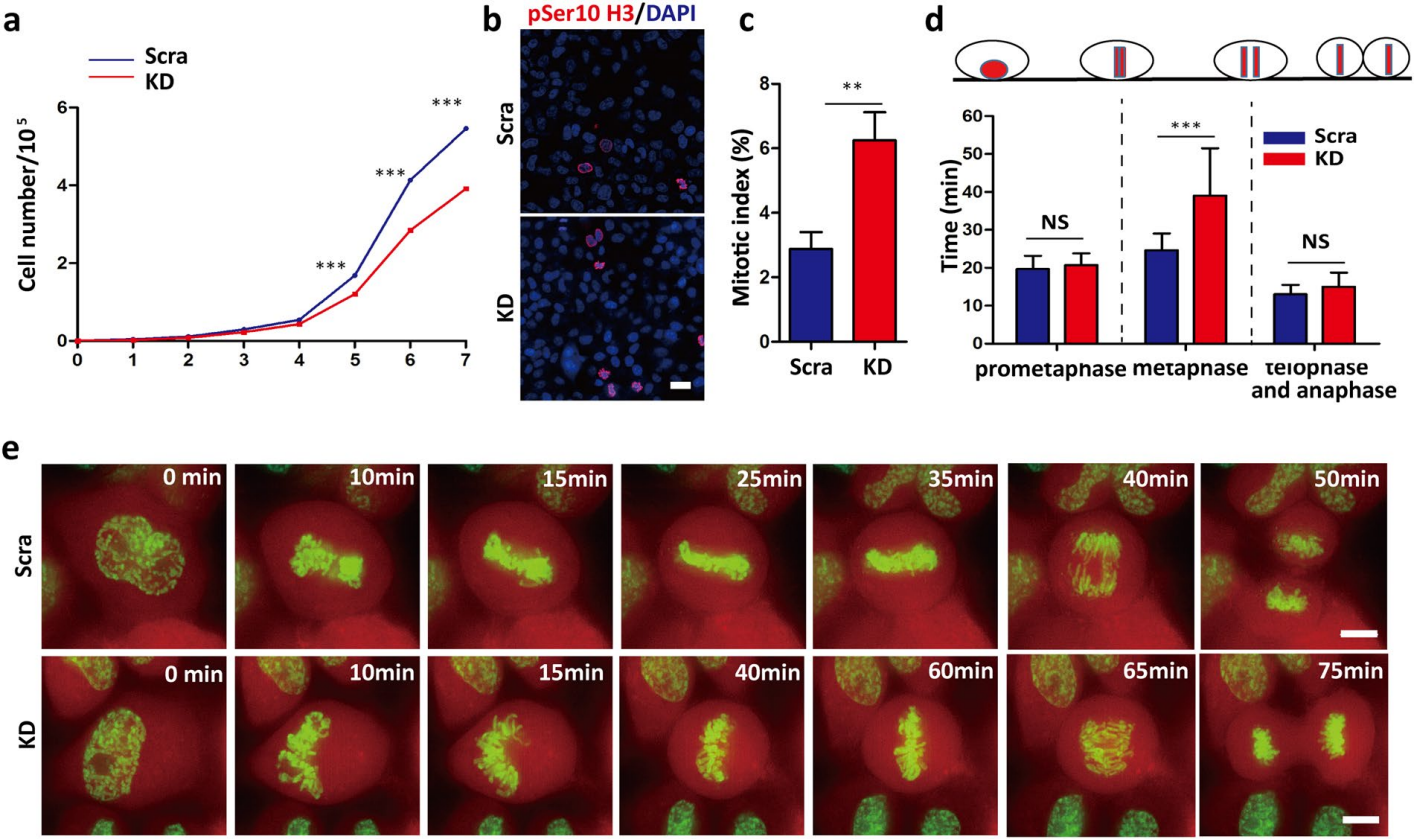
PALLD regulates mitotic progression. (**a**) Proliferation of scramble and KD cells were monitored from day 1 to day 7 (n = 3). (**b** and **c**) MI was calculated in non-synchronized HeLa cells (randomly counted the number of pSer10 H3-positive cells from 130–200 cells/slide, from three independent experiments). pSer10 H3 red, DAPI blue, scale bar 25 µm. (**d** and **e**) H2B–EGFP (green)-expressing scramble and KD cells were monitored in real time by time-lapse imaging, and the duration of mitotic phases was calculated (total randomly counted 15 division cells each group, from four independent experiments). H2B–EGFP signal and cytoplasm are green and red, respectively. Scale bar, 5 µm. ***P < 0.001; **P < 0.01 in t-test for each graph.

To investigate the roles of PALLD in mitosis, we knocked it down in HeLa cells (KD cells) using shRNAs (Supplementary Fig. 1a). Analysis of growth curves showed that average total cell number at seventh day decreased significantly in the KD group compared with the control (scramble 5.46 × 10^5^ ± 0.13 × 10^5^ *vs*. KD 3.92 × 10^5^ ± 0.12 × 10^5^, P < 0.001; average inhibition rate, 28.21%) (Fig. 1a). This finding indicated that *PALLD* depletion impaired cell proliferation in HeLa cells. To study the underlying mechanism, we calculated their mitotic index (MI). Average MI increased from 2.88% ± 0.53% to 6.25% ± 0.87% upon *PALLD* knockdown (scramble *vs*. KD, P < 0.01) **(**Fig. 1b,c). Consistent with this result, post-synchronization cell cycle analysis revealed that the transition from G2/M to G1 phase was delayed in KD cells (P < 0.05) (Supplementary Fig. 1b,c). No obvious difference in the rate of apoptosis was observed (Supplementary Fig. 1d). We further monitored the mitosis of H2B–EGFP-expressing cells in the scramble and KD groups in real time using high-resolution time-lapse microscopy. We found that *PALLD* knockdown significantly prolonged cell division time at the metaphase (scramble 24.67 ± 4.42 min *vs*. KD 39.00 ± 12.98 min, P < 0.001; average duration of metaphase prolonged by 58.09%) **(**Fig. 1d,e and Supplementary Videos 1 and 2). These data indicated that PALLD could regulate mitotic progression and cell proliferation.

### PALLD is a MT-associated protein

**Figure 2:**
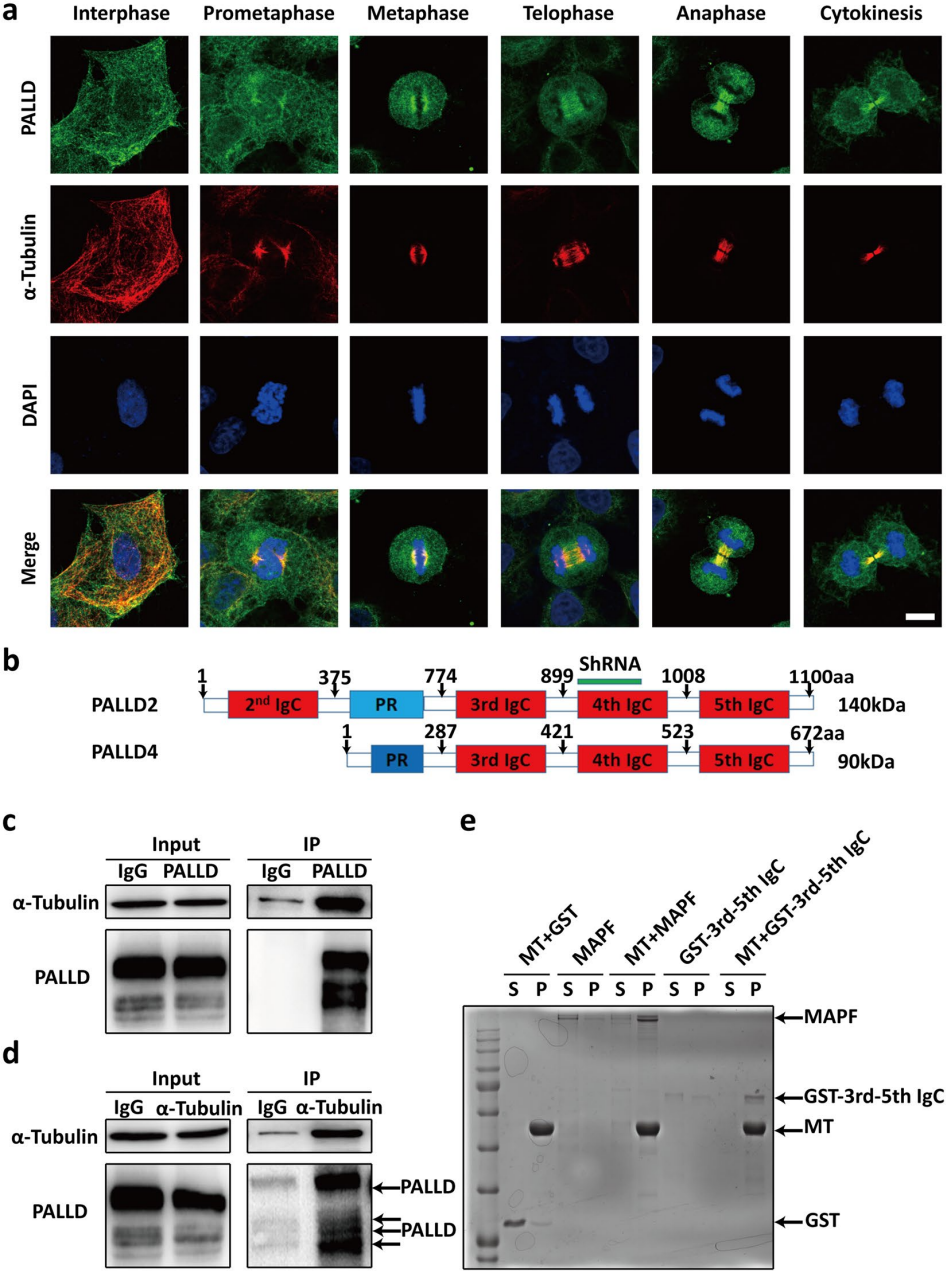
PALLD is associated with α-tubulin localizing at mitotic spindles. (**a**) Co-localization of PALLD and MTs in HeLa cells was displayed. PALLD green, α-tubulin red, DAPI blue, scale bar 10 µm. (**b**) Schematic representations of PALLD are shown, and the truncated PALLD﻿s were constructed as indicated in Figure. PR, proline-rich region; IgC, immunoglobulin C2 type domain. (**c** and **d**) Detection of the interaction between endogenous PALLD and α-tubulin in HeLa cells. (**e**) *In vitro* MT co-sedimentation of GST-tagged third to fifth IgC domain was performed. The MT-associated protein MAPF was used as positive control. The original Western blot files are presented in Supplementary Figure 13.

Next, we investigated how PALLD affects mitosis. PALLD was localized at mitotic spindles from the pro-metaphase to cytokinesis in HeLa cells (Fig. 2a). In addition, it was localized at the mitotic spindles in the metaphase of MDA-MB-231, a breast cancer cell line (Supplementary Fig. 2a). Therefore, the association of PALLD with mitotic spindles was common in different cell types. As a known microfilament-associated protein, PALLD was also localized at the cell cortex in HeLa cells (Supplementary Fig. 2b). *PALLD2* and *PALLD4* (two splice variants of *PALLD*) were constructed by one proline-rich region and immunoglobulin C-type domains (Fig. 2b). Endogenous PALLD **(**Fig. 2c,d) and overexpressed PALLD2 and PALLD4 could interact with α-tubulin via the third IgC domain (Supplementary Fig. 3a–c) in Hela cells. MT co-sedimentation assay showed that the GST-tagged third to fifth IgC domain of PALLD was able to co-pellet with polymerized MTs *in vitro* (Fig. 2e). Thus, our data demonstrated that PALLD is a novel MT-associated protein.

### PALLD sustains proper spindle orientation

**Figure 3:**
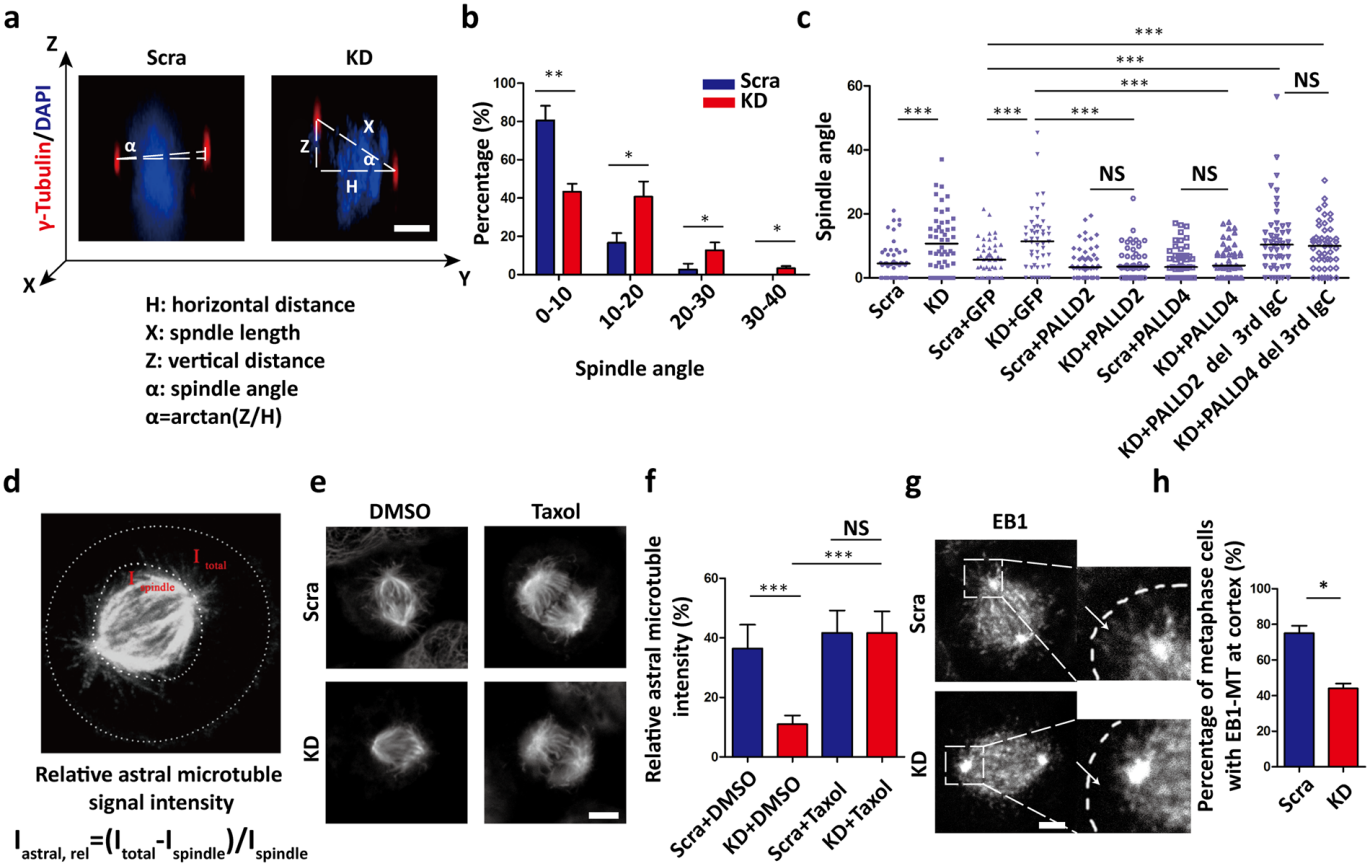
PALLD sustains proper spindle orientation. (**a**) After staining with anti-γ-tubulin (red) and DAPI (blue), image stacks of mitotic HeLa cells were acquired at 0.5 µm interval along the z-axis. The spindle angle was calculated using an inverse trigonometric function. (**b**) Distribution of spindle angles in each group was assessed (n = 50, from three independent experiments). (**c**) Median of spindle angles was calculated in scramble and KD cells, scramble and KD cells expressing an empty vector and shRNA-resistant PALLD2 or PALLD4, and KD cells expressing PALLD2 or PALLD4 with deletion of the third IgC domain (n = 50). (**d–f**) The mean relative astral MT intensity was calculated as depicted in the schematic overview in (**d**) in both groups after DMSO or 1 µM Taxol treatment (n = 20) (**e** and **f**). α-Tubulin white, scale bar 5 µm. (**g** and **h**) Mitotic HeLa cells were stained with anti-EB1 (white) (**g**), and the percentage of cells with cortical anchoring of astral MTs (**h**) was counted (n = 50, from two independent experiments). ***P < 0.001; **P < 0.01; *P < 0.05; NS, P > 0.05. Mann–Whitney U test was used to compare the median of spindle angles, whereas t-test was used for other graphs.

Furthermore, we found evidence of various mitotic defects caused by *PALLD* knockdown, including spindle tilt, off-centered spindle, and multipolar spindle at the metaphase and misaligned chromosome at the pro-metaphase. However, the abnormalities were not increased by *PALLD* knockdown at the anaphase or cytokinesis such as lagging chromosomes or chromosomal bridges (Supplementary Fig. 4).We focused on studying spindle tilt among these mitotic defects because it showed the greatest change (scramble 19.33% ± 7.57% *vs*. KD 56.67% ± 4.16%, P < 0.01, n = 50; three independent experiments). Spindle angle was calculated in 50 metaphase cells in each group, and KD cells exhibited lower percentage of spindle angle within 10° (scramble 80.67% ± 7.57% *vs*. KD 43.33% ± 4.16%, P < 0.01) and larger spindle angle (median of scramble 4.48° *vs*. KD 10.74°, P < 0.001) **(**Fig. 3a–c **)**. Spindle misorientation caused by *PALLD* knockdown was completely rescued by shRNA-resistant *PALLD2* or *PALLD4* **(**Fig. 3c **)**. As U2OS (osteosarcoma cell line) is recognized as another classic model for mitosis research, we performed spindle orientation assay and confirmed that *PALLD* depletion also led to spindle misorientation in U2OS cells (Supplementary Fig. 5). Thus, our results demonstrated that PALLD plays an important role in sustaining proper spindle orientation.

As the instability of astral MTs could contribute to spindle misorientation, we compared the levels of astral MTs between the scramble and KD cells. Our data showed that *PALLD* depletion decreased astral MTs by 69.90% (scramble 36.45% ± 8.00% *vs*. KD 10.97% ± 2.96%, P < 0.001), and loss of astral MTs could be rescued by a MT-stabilizing drug Taxol **(**Fig. 3d–f **)**. At the same time, instability of astral MTs caused by *PALLD* depletion could be completely rescued by shRNA-resistant *PALLD2* or *PALLD4* (Supplementary Fig. 6a). Furthermore, we treated HeLa cells with Taxol (1 µM) or Nocodazole (10 ng/mL) to stabilize or depolymerize astral MTs, respectively. We found that the spindle tilt in KD cells could be rescued by Taxol, whereas spindle tilt in scramble cells was increased similar to that in KD cells by Nocodazole (Supplementary Fig. 6b). These results showed that PALLD could maintain proper spindle orientation by stabilizing astral MTs.

To visualize the MT plus ends, we performed MT plus end-binding protein EB1 staining in HeLa cells^19–21^. Our data showed that EB1 tracked MT plus end of spindle MTs and astral MTs connected to the cell cortex in scramble cells. By contrast, EB1 was present at the tips of spindle MTs in KD cells, and fewer cells showed EB1 signal near the cell cortex (scramble 75.00% ± 4.24% *vs*. KD 44.00% ± 2.83%, P < 0.05) **(**Fig. 3g,h). Similarly, dynein/dynactin complex mediated the connection between astral MTs and cell cortex^22^. Our data showed that significantly less dynein (scramble 62.67% ± 6.43% *vs*. KD 37.33% ± 7.57%, P < 0.05)/dynactin (P150^Glued^, scramble 67.33% ± 3.06% *vs*. KD 32.00% ± 5.29%, P < 0.001) complex was distributed on the cell cortex in *PALLD* KD group (Supplementary Fig. 7). In addition, *PALLD* knockdown did not affect centrosome nucleation and MT regrowth in mitotic HeLa cells (Supplementary Fig. 8). Taking this into account, our results indicated that *PALLD* knockdown led to loss of connection between astral MTs and cell cortex.

### PALLD interacts with AKT1 via the third IgC domain to maintain AKT1-GSK3β activation and spindle orientation

**Figure 4:**
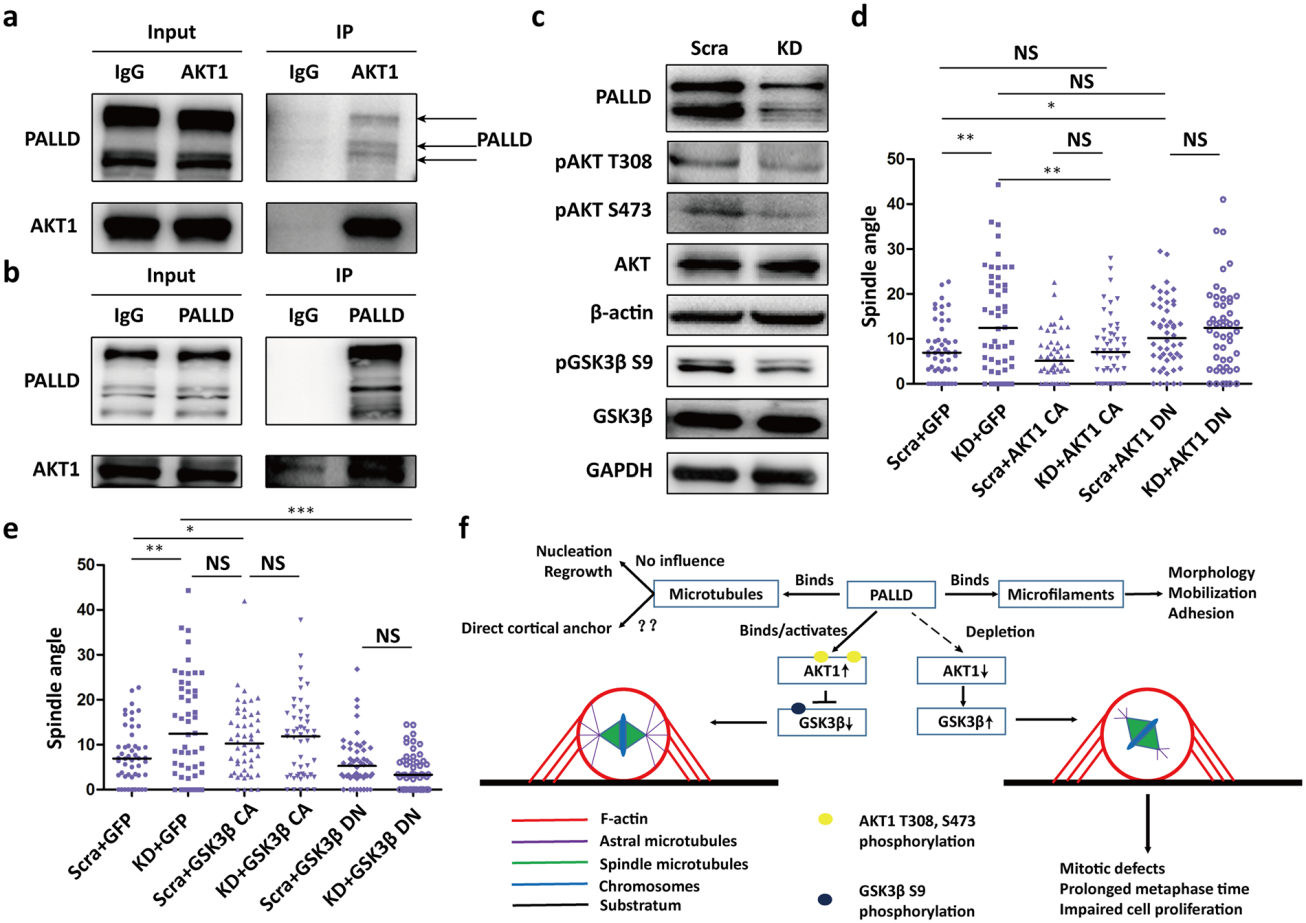
PALLD interacts with AKT1 to maintain AKT1-GSK3β activation and spindle orientation. (**a** and **b**) Results of co-immunoprecipitation between endogenous AKT1 and PALLD in mitotic HeLa cells. (**c**) After synchronization using double Thymidine, HeLa cells in the scramble and KD groups were collected, and proteins were extracted. Levels of proteins in the AKT1–GSK3β pathway were examined by Western blot. (**d** and **e**) Spindle angles of scramble and KD cells overexpressing AKT1 CA, AKT1 DN, GSK3β CA, or GSK3β DN were analyzed to determine the extent of rescue of spindle misorientation caused by *PALLD* knockdown (n = 50). (**f**) Molecular model illustrating the mechanism by which PALLD regulates spindle orientation and mitotic progress. ***P < 0.001; **P < 0.01; *P < 0.05; NS, P > 0.05. Mann–Whitney U test was used to compare median spindle angles across experimental conditions. The original Western blot files are presented in Supplementary Figure 13.

AKT1 can regulate astral MT intensity in HeLa cells^23^ and spindle orientation in early *Drosophila melanogaster* embryos^24^. We confirmed that PALLD, including PALLD2 and PALLD4, was associated with AKT1 via the third IgC domain in HeLa cells (Fig. 4a,b and Supplementary Fig. 9). In addition, the third IgC domain in PALLD had a high-affinity binding with AKT1 to support their direct interaction (Supplementary Fig. 10). Without the third IgC domain, AKT1 binding to PALLD was abrogated and led to spindle misorientation in PALLD KD cells not being rescued (Fig. 3c). After cell synchronization to M phase, protein expression was detected by Western blot. The results showed that *PALLD* deletion caused down-regulation of pAKT T308 and S473 levels in mitotic HeLa cells (Fig. 4c), indicating that PALLD could regulate AKT1 activation at the metaphase. Next, our data exhibited that the AKT inhibitor MK2206 or LY294002 significantly increased spindle angle in scramble cells, but no obvious change was observed in KD cells (Supplementary Fig. 11). Furthermore, *PALLD* knockdown induced spindle misorientation, and astral MT instability could be rescued by constitutive overexpression of activated AKT1 (myristoylated AKT1, AKT1 CA). Meanwhile, control cells with dominant negative AKT1 (AKT1 T308A and S473A, AKT1 DN) overexpression showed similar phenotypes as KD cells **(**Fig. 4d and Supplementary Fig. 12a). Thus, these data demonstrate that PALLD sustained correct spindle orientation by maintaining AKT1 activity.

AKT1 mediates Ser9 phosphorylation of GSK3β, which inhibits GSK3β and promotes MT anchoring to the periphery in interphase via regulation of APC and CLASP2 phosphorylation^25,26^. Unexpectedly, *PALLD* depletion down-regulated the pGSK3β Ser9 level in mitotic HeLa cells **(**Fig. 4c). Random spindle angles or weakened astral MTs were induced by constitutively overexpressed GSK3β (GSK3β K85R, GSK3β CA) in scramble cells, and their phenotype was similar to that in KD cells. These *PALLD* knockdown-induced abnormal phenotypes could be rescued by overexpressing dominant negative GSK3β (GSK3β S9A, GSK3β DN) (Fig. 4e and Supplementary Fig. 12b). Thus, our data showed that PALLD maintains AKT1–GSK3β pathway activation to stabilize astral MTs and sustain proper spindle orientation (Fig. 4f).

## Discussion

As an actin-binding and microfilament-associated protein, PALLD has been reported to regulate cell morphology, mobilization, adhesion, invasion, and metastasis of cancer cells^27–30^. Surprisingly, our data showed that PALLD also is a novel MT-associated protein and plays an important role in cell proliferation and mitosis. Knockdown *PALLD* in HeLa cells led to mitotic defects and metaphase extension. Consistently, MI increase and transition delay from M to G1 were observed in KD cells. Mitotic defects, such as multipolar spindle, could induce cell apoptosis^19^, but we did not find significant difference in cell apoptotic rate between the *PALLD* KD and control groups. The reason might be that the percentage of cells with multipolar spindle was low, and self-adjustment was not disturbed significantly. Therefore, cells without PALLD can overcome these defects by increasing division time.

In our study, *PALLD* knockdown-induced mitotic defects occurred mainly at metaphase, so *PALLD* depletion significantly prolonged the duration of metaphase. Among those mitotic defects, we focused on studying spindle tilt. Proper spindle orientation determines well-oriented cell division, which is critical for cell fate specification, tissue homeostasis, and tissue organization^3,31^. Spindle orientation is mainly determined by astral MT stability, F-actin integrity, and cell cortical complex distribution^3^. We found that *PALLD* knockdown induced astral MT instability. The evidence includes high-frequency spindle tilt and off-centered spindles occurred. In addition, stabilizing astral MTs by Taxol rescued spindle misorientation due to *PALLD* depletion. Astral MT stability depends on MT nucleation at the centrosome, MT dynamics, MT–cortex interactions, and MT behavior at the cortex^4^. We further found that PALLD depletion impaired the connection between astral MTs and the cell cortex. Therefore, we identified PALLD as a new regulator of spindle orientation.

Our results showed that the third IgC domain of PALLD was required for its interaction with MTs. It also mediated its interaction with F-actin^14,32,33^. The interaction between MT and microfilament cytoskeleton exists at the cell cortex and around mitotic spindles^34,35^. However, we do not know whether simultaneous or competitive binding to PALLD exists between MTs, F-actin, and other proteins. Therefore, we presumed that PALLD plays different roles through interacting the third IgC domain with different partners.

Furthermore, we found that AKT1 was associated with PALLD, and its activity was reduced after *PALLD* depletion. In turn, the inhibitory effect on GSK3β was lost in mitotic HeLa cells. AKT1 phosphorylates PALLD at Ser507 to regulate its actin-bundling function^36,37^. Meanwhile, our results showed that PALLD could also regulate AKT1 phosphorylation at Thr308 and Ser473 to maintain its activation in the metaphase. Several groups have reported that mitosis-specific AKT phosphorylation levels were up-regulated^23,38^. AKT is mainly activated by PI3K–PDK1, but many cell signaling molecules, such as Src, ERK, MEK, or PKC, also crosstalk with this central axis^39^. We presumed that PALLD might promote the interaction between AKT1 and its upstream molecules as a scaffold protein and might help anchor AKT1 to proper subcellular structures for its phosphorylation. AKT1 was reported to regulate spindle orientation via inhibiting GSK3β functions^24,40^. Inactivation of GSK3β leads to the accumulation of APC and CLASP2 at the plus end of MTs, helping them interact with microfilaments at the cell cortex and making them more stable^25,26^. Therefore, AKT1 and GSK3β also can function as astral MT stabilizers through promoting their cell cortical anchorage. In our studies, *PALLD* depletion-induced spindle misorientation can be rescued by AKT1 CA or GSK3β DN. Therefore, AKT1–GSK3β might be the major downstream pathway of PALLD for sustaining proper spindle orientation, but the detailed mechanisms of how PALLD regulated AKT1 activation need to be further investigated in future.

Interestingly, PALLD regulates spindle orientation acting as a novel MT-associated protein and modulator of the AKT1–GSK3β pathway, which was quite different from known regulators^2,38,41–47^. PALLD had no influence on MT nucleation or regrowth in HeLa cells although it bound to MTs. Whether PALLD could directly control MTs anchoring to the cell cortex still needs to be investigated. We speculated that PALLD is a linker of the AKT1–GSK3β pathway and MTs. It might be a new class of spindle orientation regulators, which anchor cytoskeleton-related kinase to the cytoskeleton.

In summary, our data demonstrated that PALLD, a novel MT-associated protein, played important roles in spindle orientation, mitotic progression, and cell proliferation. These activities were mainly mediated by the interaction between PALLD and AKT1 in mitotic cells, which maintained activation of the AKT1–GSK3β pathway and stabilized astral MTs to sustain proper spindle orientation.

## Methods

### Cell culture, cell cycle synchronization, and reagents

HeLa, MDA-MB-231, U2OS, and 293 T cells were supplied with DMEM (Gibco) containing 10% fetal bovine serum (HyClone), 100 U/mL penicillin, and 100 mg/ml streptomycin (Gibco) in a 5% CO_2_ humidified incubator at 37 °C. To synchronize cells in M phase, HeLa cells were blocked with 100 ng/mL Nocodazole (Sigma) for 16 h or treated with 2 mM Thymidine (Sigma) for 16 h and released to Thymidine-free medium for 10 h, followed by second exposure to 2 mM Thymidine for 16 h before being released for 9 h. The following reagents were also used: Taxol (1 µM, Selleck), LY294002 (50 µM, Selleck), and MK2206 (40 µM, Selleck).

### Plasmid construction, transfection, and stable cell lines

The scramble (targeted sequence: AGCGTGTAGCTAGCAGAGG) and *PALLD* shRNA (targeted sequence: TGGAGATCTGACTGTTCAA) sequences were cloned into the BamH I–EcoR I sites of the pLVX-ShRNA2 vector. PALLD2 and PALLD4 were amplified from the cDNA library and cloned to the pPBCAG vector at Afl II–Pme I sites. Different truncated forms of *PALLD* representing different domains were amplified from plasmids pPBCAG-PALLD2 and pPBCAG-PALLD4 and then cloned into pPBCAG vector as shown above. ShRNA-resistant *PALLD* variants were constructed by site-directed mutagenesis. In addition, the third to fifth IgC domain and full length of *PALLD4* were also cloned into BamH I–Xho I sites of the pGEX-4T2 vector for protein purification *in vitro*. All products were confirmed by DNA sequencing. HeLa cells and 293 T cells were transfected with Lipofectamine 2000 (Invitrogen) and the Calcium Phosphate Cell Transfection Kit (Beyotime), respectively. HeLa cells with stable knockdown of *PALLD* were made by transfecting with lentivirus-expressing *PALLD* ShRNA, which were selected by flow cytometry-based sorting. Knockdown efficiency was determined by Western blot.

### Cell proliferation, apoptosis, and cell cycle analysis

To monitor the proliferation of HeLa cells, 1000 cells of each group were placed into a 96-well plate. After four days in culture, cells in each well were passaged at 1:10 ratio because of limited culture surface. Afterward, 1:10 volume of CCK-8 (Dojindo) was added to the medium and cultured for 2 h at 37 °C. The absorbance was tested at 450 nm, whereas the standard curve of HeLa cells was measured to convert absorbance value to cell counts.

For detecting the apoptotic rate of cells, cells were collected after treatment and washed twice in 1 × PBS. Following staining with Annexin-V FITC antibody (Biotool) for 10 min, cells were subjected to flow cytometry analysis.

For cell cycle analysis, cells were collected after synchronization and then fixed with 75% ethanol at −20 °C for 24 h. After washing with 1 × PBS once, 100 µg/mL RNase and 100 µg/mL PI were added in turn for 30 min each. The cells were then subjected to flow cytometry for cell cycle analysis.

### Immunoblots

Cells were collected and extracted using lysis buffer (50 mM Tris-HCl (pH 7.4),150 mM NaCl, 1% Triton X-100, 1% sodium deoxycholate, 0.1% SDS) to which protease inhibitor cocktail (Roche) and PhosSTOP (Roche) tablets had been added. The lysates were centrifuged at 15,600 × g for 10 min, and the supernatants were collected for Western blot after quantification of protein concentration by Bradford assay (Beyotime). The following antibodies were applied: anti-Palladin (Proteintech), anti-α-tubulin (Sigma, Proteintech), anti-Akt (Cell Signaling), anti-phospho-Thr308-Akt (Cell Signaling), anti-phospho-Ser473-Akt (Cell Signaling), anti-GSK3β (Proteintech), anti-phospho-Ser9-GSK3β (Cell Signaling), anti-β-actin (Sigma), anti-FLAG tag (Abmart, Cell Signaling), and anti-HA tag (Cell Signaling).

### Co-immunoprecipitations

For co-immunoprecipitation of endogenous proteins, HeLa cells were lysed with a buffer containing 50 mM Tris-HCl (pH 7.4), 150 mM NaCl, 1 mM EDTA, 1% Triton X-100, protease inhibitor cocktail (Roche), and PhosSTOP (Roche) tablets. Subsequently, the supernatants were collected after centrifugation at 15,600 × g for 10 min. After incubation with 1–2 µg primary antibody for 6 h at 4 °C, 20 µL protein A/G agaroses (Santa Cruz) were added and incubated for another 2 h. The immunoprecipitates were washed with 1 × PBS four times and eluted using 2 × SDS sample buffer (125 mM Tris-HCl, pH 6.8, with 4% SDS, 20% (v/v) glycerol, and 0.004% bromophenol blue) containing 5% DTT. For co-immunoprecipitation of overexpressed FLAG-tagged proteins, the supernatant extracts were incubated with 20 µL Anti-FLAG Affinity Gel for 3 h and then eluted in 100 µL 3 × FLAG peptide (150 ng/µL) after washing. The final products were then subjected to detection by Western blot.

### Immunofluorescence staining

For detecting PALLD, cells were fixed with 3.7% formaldehyde at 37 °C for 10 min, followed by 0.5% Triton-X 100 in PBS at 37 °C for 10 min. To display α-tubulin, γ-tubulin, P150^Glued^, and dynein heavy chain staining, cells were incubated with methanol at −20 °C for 20 min. Cells were blocked with 3% BSA for 1 h and then incubated at 4 °C overnight with the following primary antibodies: Rabbit anti-Palladin (1:50, Proteintech), Mouse anti-α-tubulin (1:500, Sigma or Abcam), and Mouse anti-γ-tubulin (1:100, Abcam or Sigma, GTU88), Rabbit anti-P150^Glued^ (1:50, Proteintech), and Rabbit anti-dynein heavy chain (1:50, Proteintech). The cells were then washed with 1 × PBS three times and incubated for 2 h with the following secondary antibodies: Alexa Fluor 488- or 594-goat anti-mouse or anti-rabbit IgG [H + L] antibodies (Invitrogen). Finally, DAPI (Invitrogen) staining was conducted for 10 min after washing.

### Time-lapse images

Scramble or KD cells with H2B–EGFP expression were plated in 3 cm glass bottom dishes. After synchronization using double Thymidine, cells were cultured in Leibovitz’s L-15 Medium (Gibco) containing 10% FBS at 37 °C, and time-lapse images were obtained using a DeltaVision OMX microscope every 5 min for 6 h.

### MT co-sedimentation assay

*In vitro* MT binding assay was performed using the Microtubule Binding Protein Spin-down Assay Kit (Cytoskeleton, BK029) following the manufacturer’s instructions. In test samples, 5 µg GST or GST-third to fifth IgC domian proteins with purity more than 95% was added. The results were analyzed with SDS-PAGE followed by Coomassie Blue staining.

### Mitotic defect calculation

For detecting multipolar spindle, misaligned chromosome, off-centered spindle position, or spindle tilt, we randomly picked 50 metaphase cells in the scramble or KD group and then calculated the percentage of these mitotic defects. For calculating the frequency of lagging chromosomes and chromosomal bridge, 50 anaphase cells were randomly picked. All data were from three independent experiments.

### Spindle orientation analysis

HeLa cells were plated on coverslips and synchronized at M phase using double Thymidine and then fixed and stained with γ-tubulin antibody and DAPI. Cells at the metaphase were selected for spindle orientation analysis by confocal imaging with 0.5 µm z-sections. Spindle angle calculation formula was α = arctan[Z/H], where the vertical (Z) and horizontal distances (H) between the two spindle poles were measured and spindle angle (α = arctan[Z/H]) was calculated using an inverse trigonometric function^2,10^.

### MT regrowth assay

HeLa cells were placed in 4 °C for 2 h to depolymerize MTs completely and then to 37 °C and fixed at 0, 2, 5, and 8 min after recovery. Co-staining of α-tubulin antibody and DAPI was displayed, and the length of MTs was analyzed^19^.

### Centrosome nucleation assay

After depolymerizing MTs completely at 4 °C for 2 h, HeLa cells were moved to 37 °C and fixed at 1.5 min after return. Centrosomes were marked by α-tubulin antibody, and their intensity was measured by ImageJ software^48^.

### Protein binding kinetics and affinities

Binding of GST-AKT1 CA (85 kDa), α-tubulin (55 kDa), or GST (25 kDa, negative control) to GST-PALLD third IgC domain (40 kDa) was tested using an Octet RED96 instrument. All proteins were dissolved in the same buffer (20 mM NaH_2_PO_4_/Na_2_HPO_4_, 50 mM NaCl, pH = 7.6). Assays were performed at 30 °C in solid black 96-well plates, and the final volume for each well was 200 µL. GST-PALLD third IgC was labelled by biotin, and streptavidin-coated sensors were used. The final concentration of GST-AKT1 CA or α-tubulin was 200, 100, 50, 25, 12.5, and 0 nM. Also, a sensor with only PALLD third IgC biotin or testing proteins was displayed for correction of baseline drift. The running protocol was set as follows: 60 s biosensor baseline, 360 s loading biotin-labelled protein, 120 s protein loaded biosensor baseline, 600 s protein association, and 180 s protein dissociation. Octet data were processed by ForteBio Data Acquisition software v 8.1. *K*_D_ values or other kinetic parameters were calculated from 1:1 global fitting for GST-PALLD third IgC domain^49^.

### Statistical analysis

For spindle orientation analysis, Mann–Whitney U test was used to compare median spindle angles between scramble and knockdown groups due to non-normal distribution of data. For other data, Student’s t-test was applied to compare the differences between the two groups. A value of P < 0.05 was considered statistically significant.

### Data availability

All relevant data supporting the findings of this study are available within the article and its Supplementary Information files, or from the corresponding author on request.

## Electronic supplementary material

Supplementary information

Supplementary Movie_1

Supplementary Movie_2
