# Supplementary material for: Palladin is a novel microtubule-associated protein responsible for spindle orientation
Source: Sci Rep. 2017 Sep 18;7:11806. doi: 10.1038/s41598-017-12051-w (PMC5603589; doi:10.1038/s41598-017-12051-w)
Supplement: Supplementary file 1 — Supplementary information [file 41598_2017_12051_MOESM1_ESM.pdf]

Supplementary information

Palladin is a novel microtubule-associated protein responsible for spindle orientation

Xiang Zhang[1]*, Xinlei Chen[1]*, Jing Liu[1]*, Xin Xu[1], Yuanliang Zhang[1], Zheng Ruan[1], Yinyin Xie[1], Qiuhua Huang[1]†, Tong Yin[1,2]†, Zhu Chen[1,2] & Saijuan Chen[1,2]†

[1]State Key Laboratory of Medical Genomics, Shanghai Institute of Hematology, Rui Jin Hospital, Shanghai Jiao Tong University School of Medicine, 200025 Shanghai, China.

[2]The National Research Center for Translational Medicine, Shanghai Jiao Tong University School of Medicine, 200025 Shanghai, China.

*These authors contributed equally to this work.

†Correspondence and requests for materials should be addressed to S.-J.C. (email: sjchen@stn.sh.cn) or T.Y. (email: yintong0101@163.com) or Q.-H.H. (email: hqh10632@rjh.com.cn).

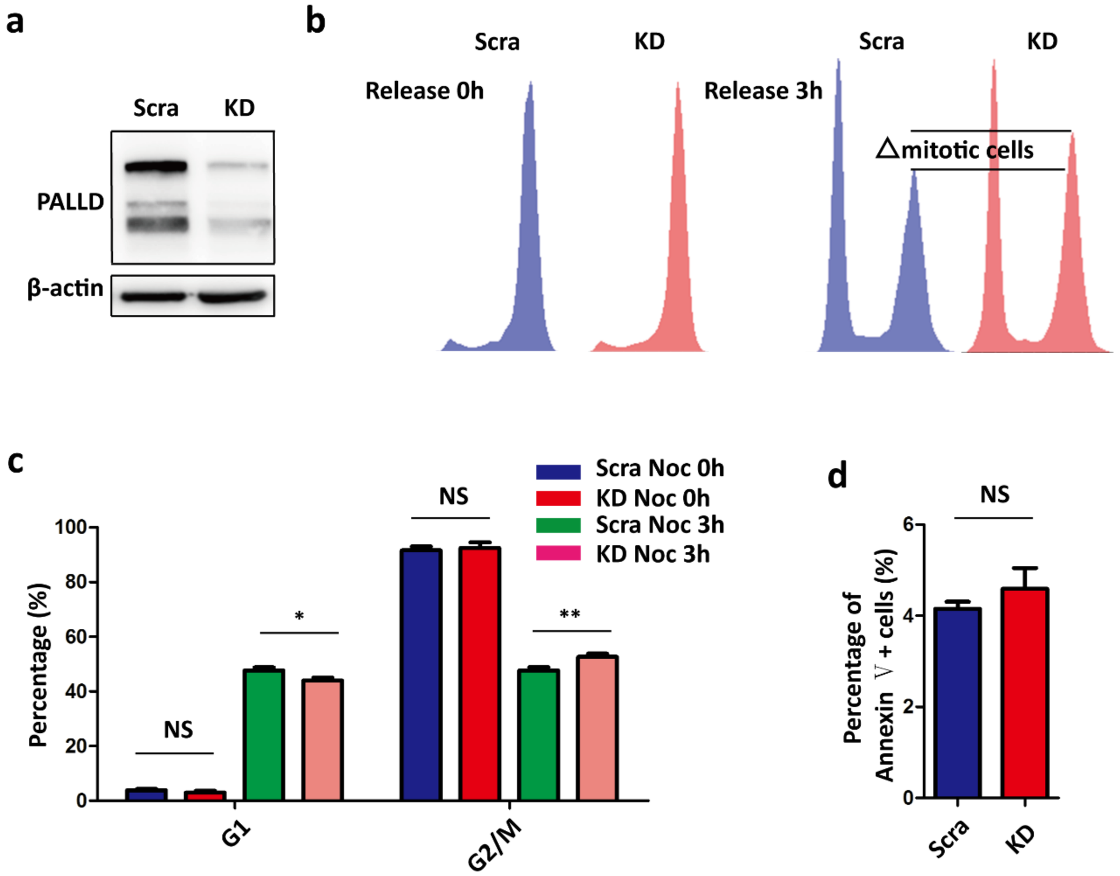

Supplementary Figure 1. *PALLD* knockdown delays transition from G2/M to G1 in HeLa cells and does not increase apoptosis. (**a**) Efficiency of shRNA-mediated *PALLD* knockdown in HeLa cells was examined by Western blot. (**b and c**) After blocking with Nocodazole and then releasing for 0 and 3 h, cells in the scramble and *PALLD* KD groups were subjected to cell cycle analysis. Summary statistics are shown (from three independent experiments). (**d**) Apoptosis rates of scramble and KD groups were detected by Annexin V staining (from three independent experiments). **, $P < 0.01$; *, $P < 0.05$; NS, $P > 0.05$. T-tests were used for assessing statistical significance.

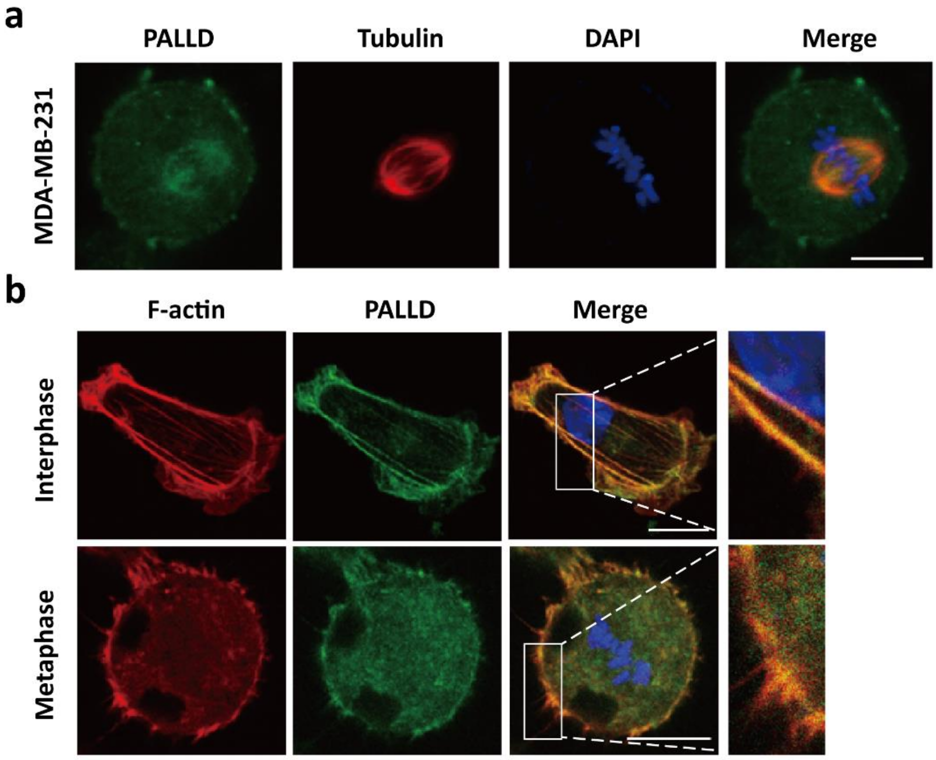

Supplementary Figure 2. PALLD localizes at mitotic spindles and cell cortex. (**a**) Co-staining of anti-PALLD (green) and anti-α-tubulin (red) was displayed in metaphase MDA-MB-231 cells. (**b**) Distribution of PALLD on F-actin was stained by anti-PALLD (green) and Alexa Fluor 546-phalloidin (red). Chromatin was stained by DAPI (blue). Scale bar, 5 µm.

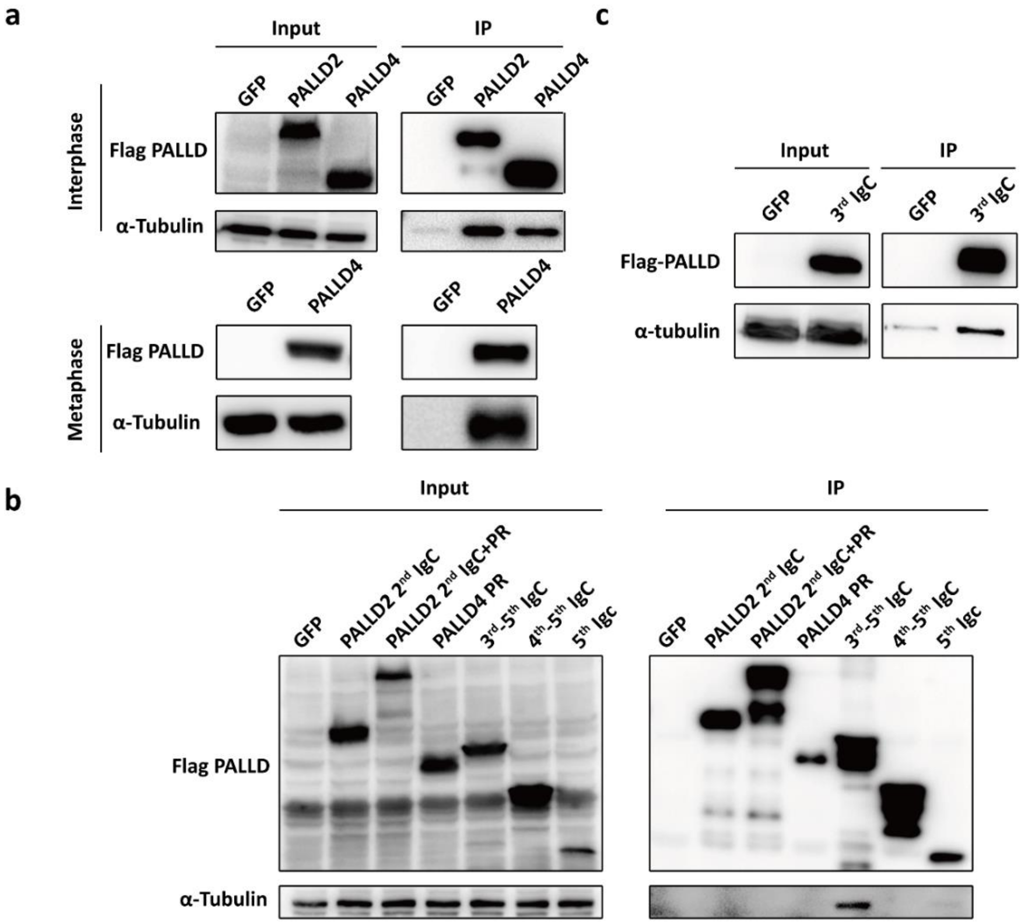

Supplementary Figure 3. PALLD interacts with tubulin via the third IgC domain. (**a**) PALLD interacted with tubulin during the metaphase and interphase. Co-IP was displayed in mitotic or non-synchronized HeLa cells with overexpressed empty vector, PALLD2, or PALLD4. (**b and c**) HeLa cells were transfected with empty vector or FLAG-tagged fragments of PALLD2 and PALLD4 (**b**) or FLAG-tagged 3rd IgC domain (**c**). Co-immunoprecipitation was conducted using Anti-FLAG Affinity Gel. The original Western blot files are presented in Supplementary Figure 13.

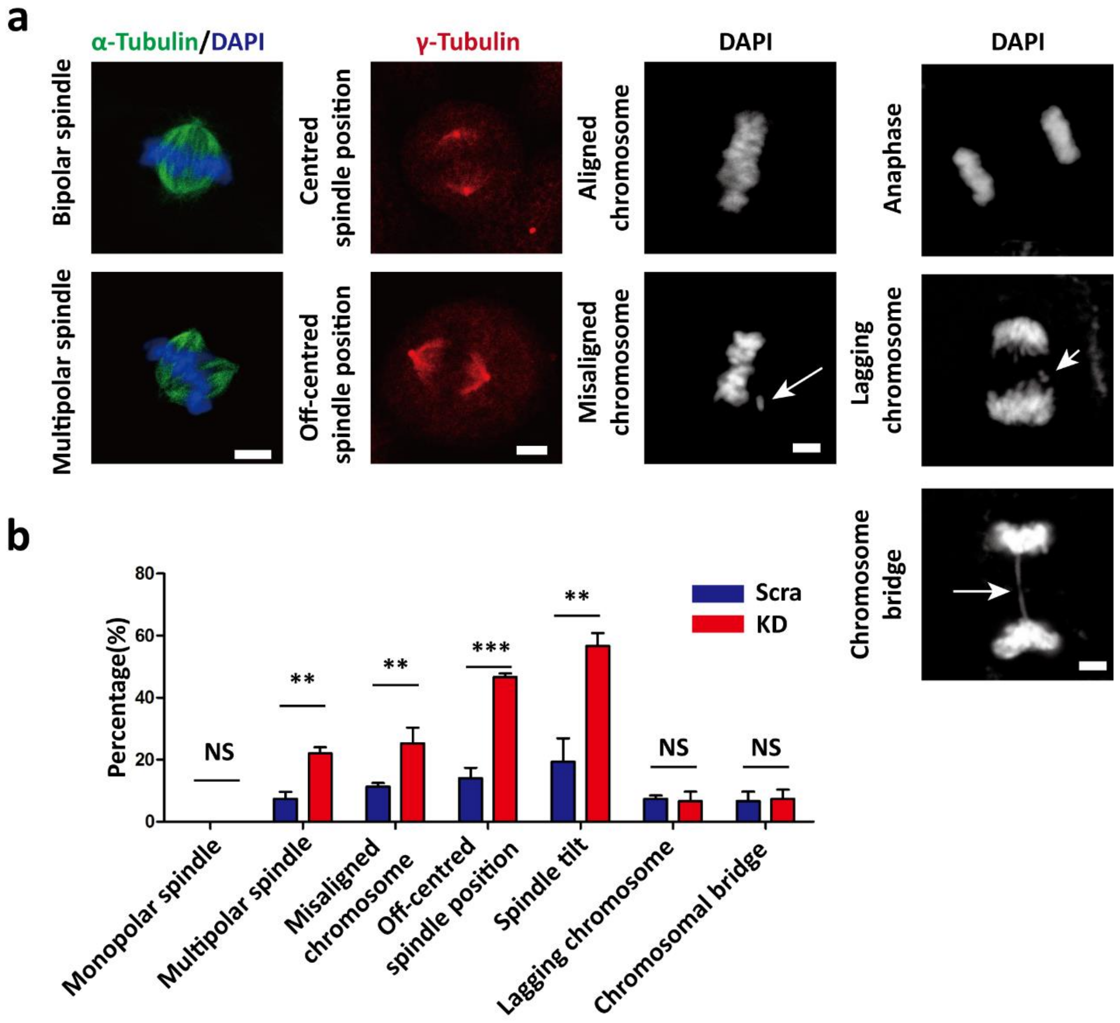

Supplementary Figure 4. ***PALLD*** **knockdown leads to mitotic defects.** (**a** and **b**) Representative pictures (**a**) and the percentage (**b**) of multipolar spindle, misaligned chromosome, off-centered spindle position, lagging chromosomes, and chromosomal bridge in scramble and KD groups are shown (mean ± SD; n = 50; from three independent experiments). α-Tubulin, green; γ-tubulin, red; DAPI, white; scale bar, 5 μm. **, P < 0.01; ***, P < 0.001.

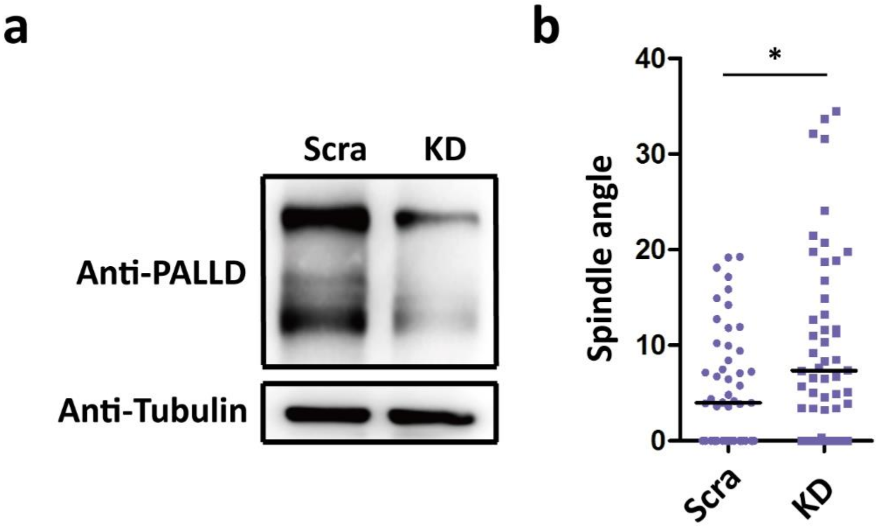

Supplementary Figure 5. ***PALLD*** **depletion leads to spindle misorientation in U2OS cells.** (**a**) Efficiency of shRNA-mediated *PALLD* knockdown in U2OS cells was examined by Western blot. (**b**) Median of spindle angles was calculated in scramble and KD cells (n = 50). *, P < 0.05. Mann–Whitney U test was used to compare the median of spindle angles.

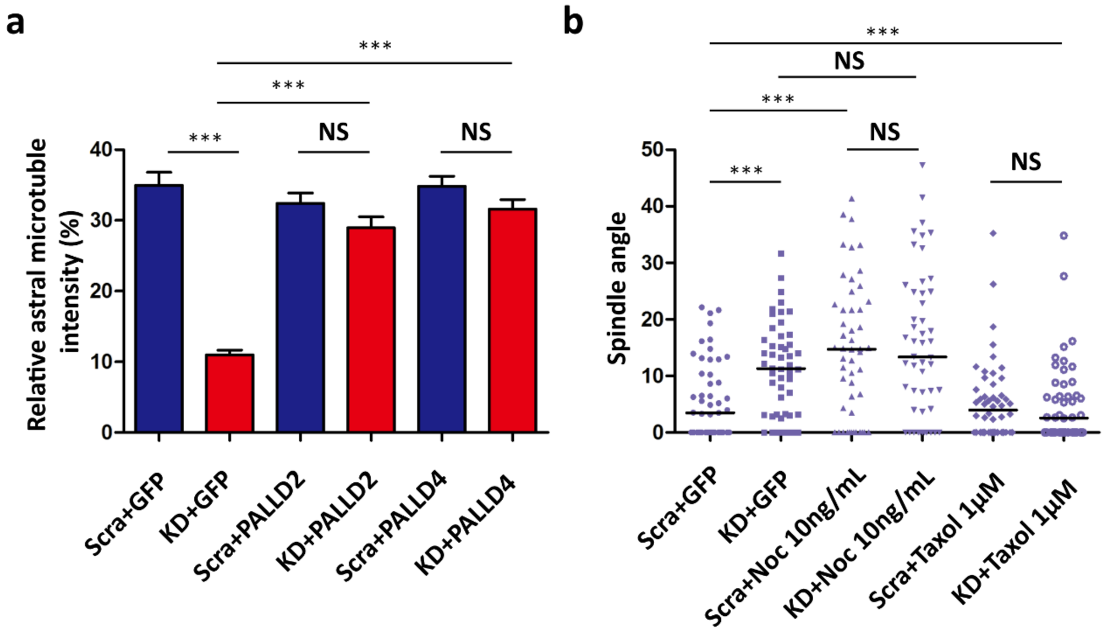

Supplementary Figure 6. PALLD stabilizes astral MTs. (**a**) Scramble and KD cells were transfected with empty vector and shRNA-resistant *PALLD2* or *PALLD4*, respectively. The relative astral MT intensity of metaphase cells was measured in each group (data shown as mean ± SEM; n = 20). (**b**) Spindle angles were measured in HeLa cells treated with DMSO, 10 ng/mL Nocodazole, or 1 μM Taxol, and the median was calculated (n = 50). ***, P < 0.001; NS, P > 0.05. Mann–Whitney U test was used to compare median spindle angles, and t-test was used for other comparisons.

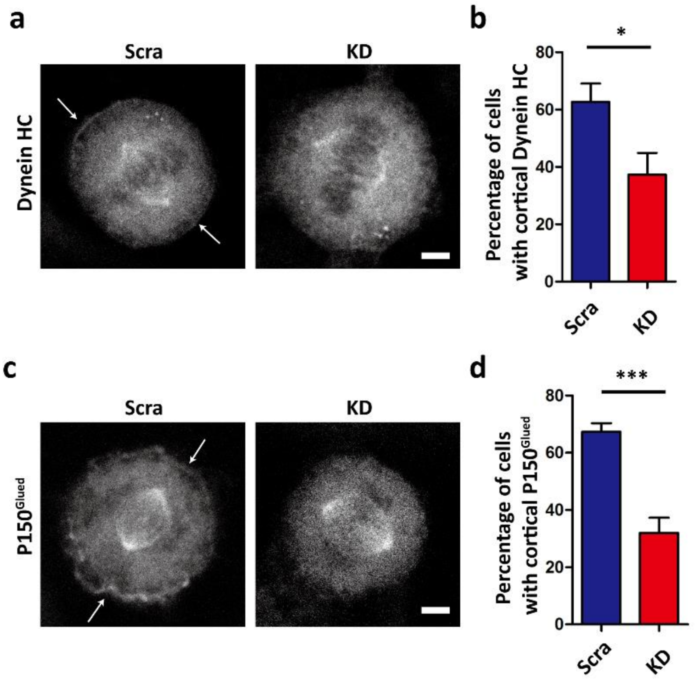

Supplementary Figure 7. *PALLD* depletion leads to defects in cortical dynein/dynactin distribution. Mitotic HeLa cells were stained with anti-dynein HC (**a**) or P150^{Glued} (**c**), and the percentage of cells with cortical dynein (**b**) or P150^{Glued} (**d**) in each group was counted (n = 50, from three independent experiments). ***, P < 0.001; *, P < 0.05. T-test was used for graphs.

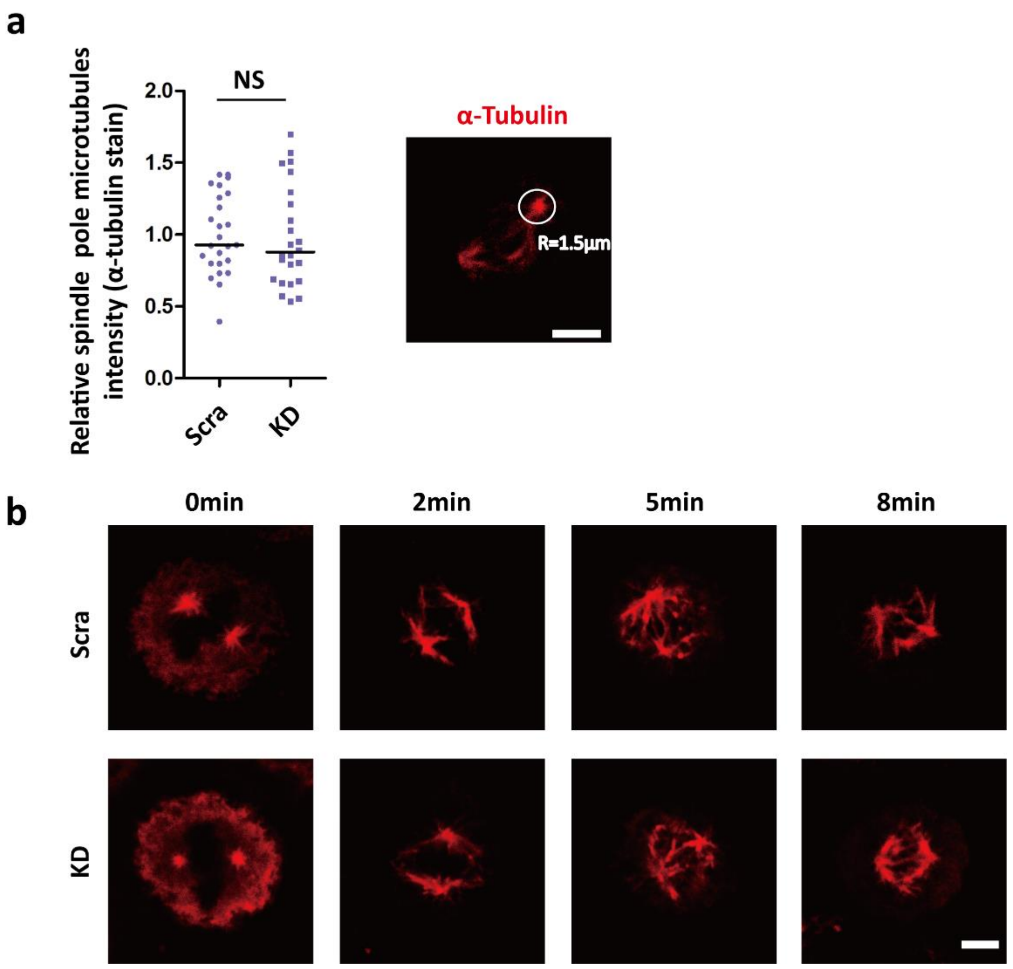

Supplementary Figure 8. *PALLD* knockdown does not affect centrosome nucleation and MT regrowth in mitotic HeLa cells. (**a**) After incubation at 4 °C for 2 h to depolymerize MTs, HeLa cells were returned to 37 °C and then fixed at 1.5 min after recovery. The fluorescence intensity of centrosomes was measured in a 1.5 µm radius of circle centered on α-tubulin as indicated in the right panel. The relative intensity was calculated by comparing the mean intensity of scramble cells (n = 25). Scale bar, 5 µm. (**b**) MT dynamics of HeLa cells were determined by α-tubulin staining (red). HeLa cells were fixed at different time points after recovery from MT depolymerization by 4 °C incubation. Scale bar, 5 µm. NS, $P > 0.05$. T-test was used for graphs.

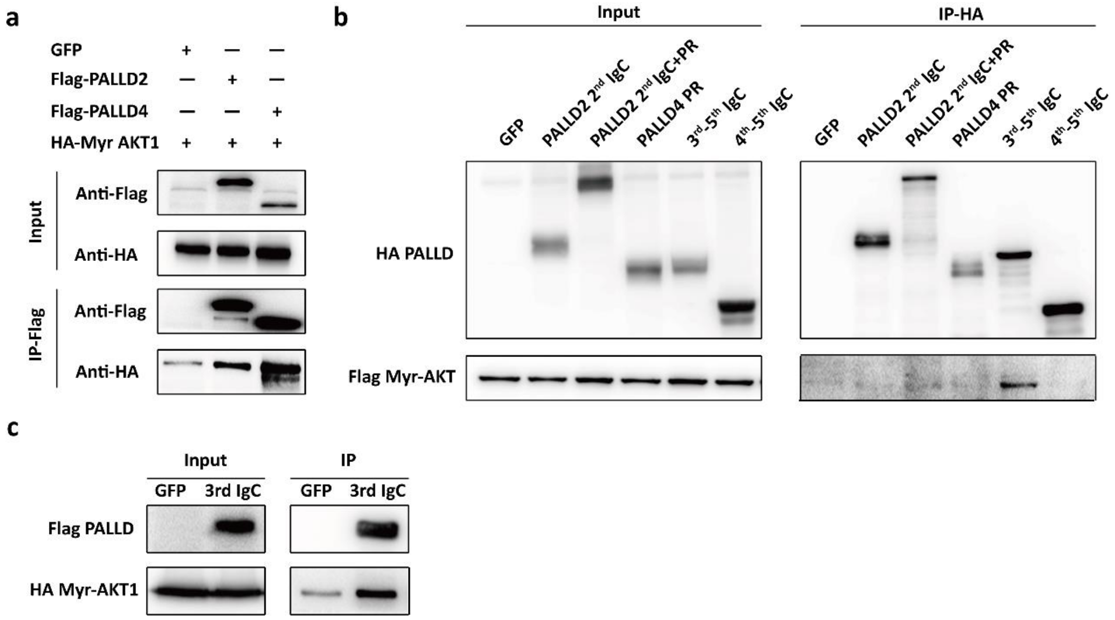

Supplementary Figure 9. PALLD interacts with AKT1 at the third IgC domain. (**a–c**) Co-IP assay was conducted in HeLa cells with overexpressed HA-tagged Myr-AKT1 and FLAG-tagged PALLD (**a**), or HA-tagged fragments of PALLD and FLAG-tagged Myr-AKT1 (**b**), or FLAG-tagged third IgC domain and HA-tagged Myr-AKT1 (**c**). The original Western blot files are presented in Supplementary Figure 13.

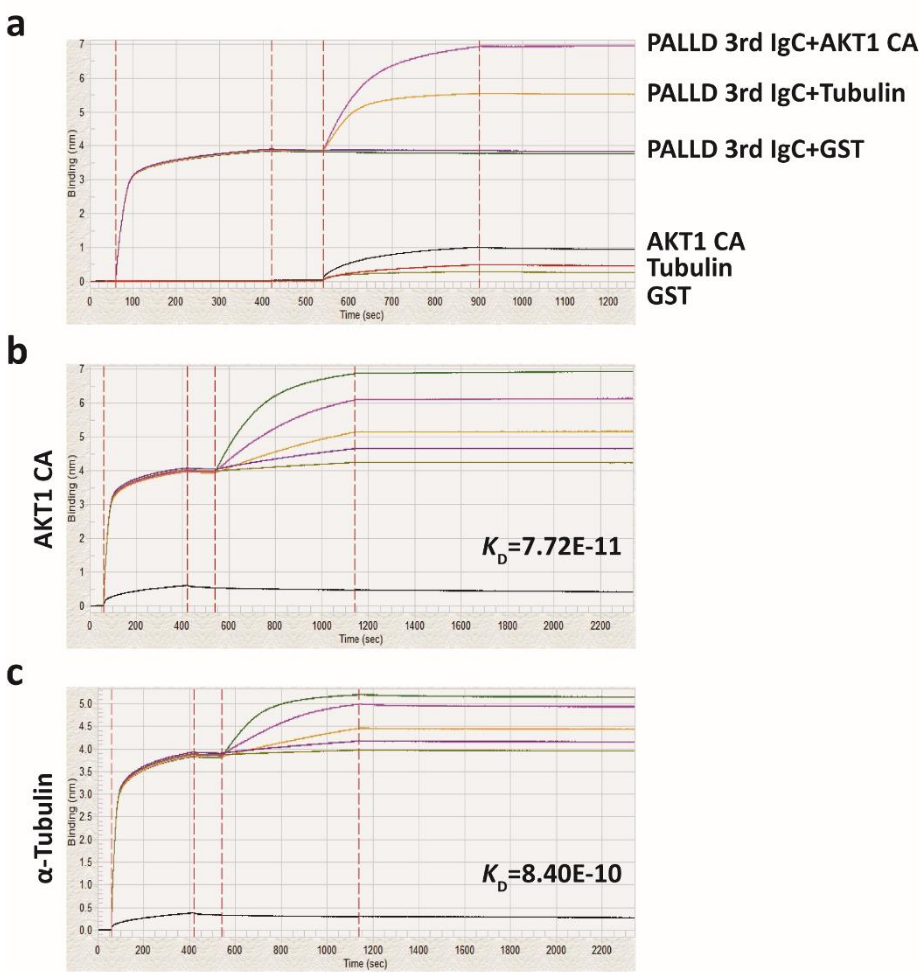

Supplementary Figure 10. PALLD third IgC domain had a higher affinity binding with AKT1 than tubulin. Octet binding of GST-AKT1 CA and α-tubulin with GST-PALLD third IgC domain was tested. GST was used as negative control (**a**). Sensorgrams were obtained from an Octet RED96 instrument using 200, 100, 50, 25, 12.5, and 0 nM for GST-AKT1 CA (**b**) or α-tubulin (**c**). K_D values were calculated from 1:1 global fitting for GST-PALLD third IgC domain.

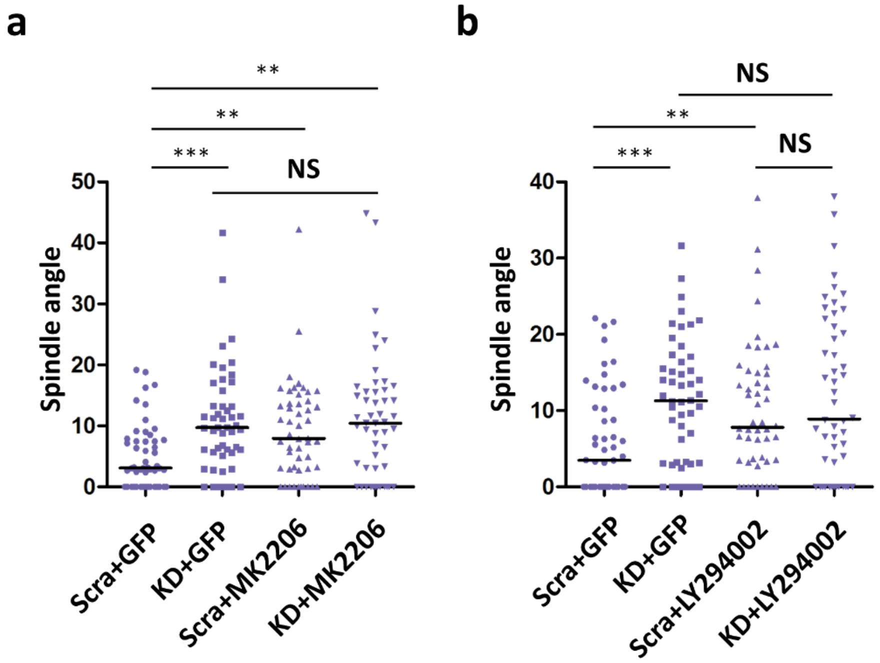

Supplementary Figure 11. Maintenance of AKT activity by PALLD is required for correct spindle orientation. (**a and b**) KD and scramble cells were treated with DMSO, MK2206 (**a**) or LY294002 (**b**). The median was calculated (n = 50). ***, $P < 0.001$; **, $P < 0.01$; NS, $P > 0.05$. Mann–Whitney U test was used to compare the median of spindle angles.

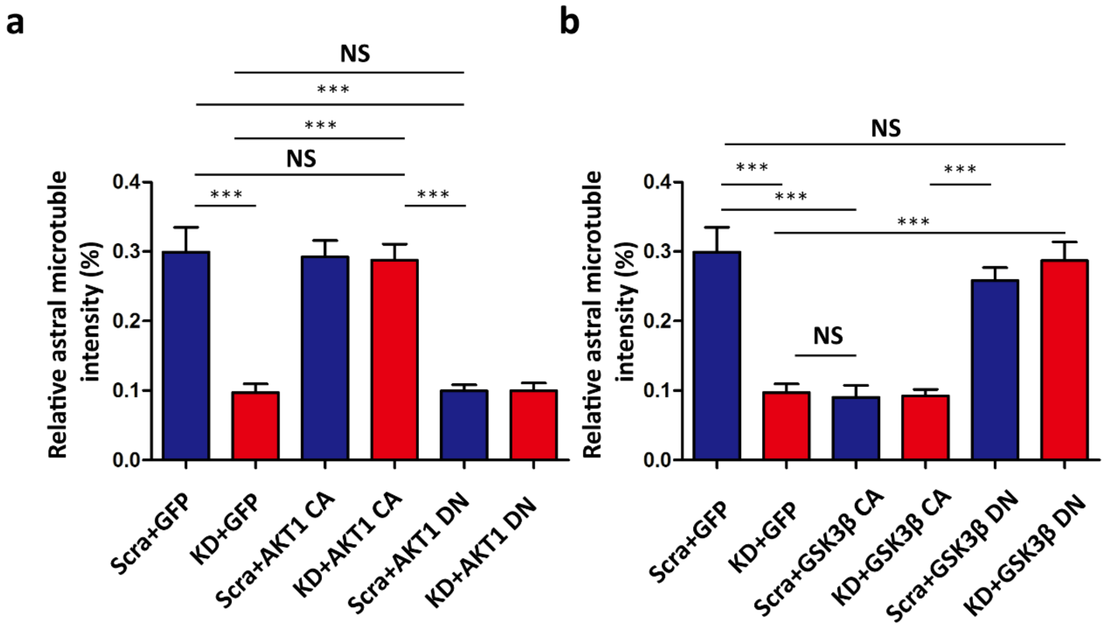

Supplementary Figure 12. PALLD maintains stability of astral MTs via the AKT1–GSK3β pathway. (**a and b**) *PALLD* knockdown-mediated astral MT instability was rescued by AKT1 CA and AKT1 DN (**a**) and GSK3β CA and GSK3β DN (**b**). Relative astral MT intensity (mean ± SEM; n = 20) was calculated 48 h after transfection. ***, $P < 0.001$; NS, $P > 0.05$. Means of astral MT signal intensity were compared by t-test.

Supplementary Videos. Live imaging of mitosis in HeLa cells. Videos showing prolonged metaphase duration in KD (1) vs. scramble (2) shRNA-treated HeLa-H2B cells.

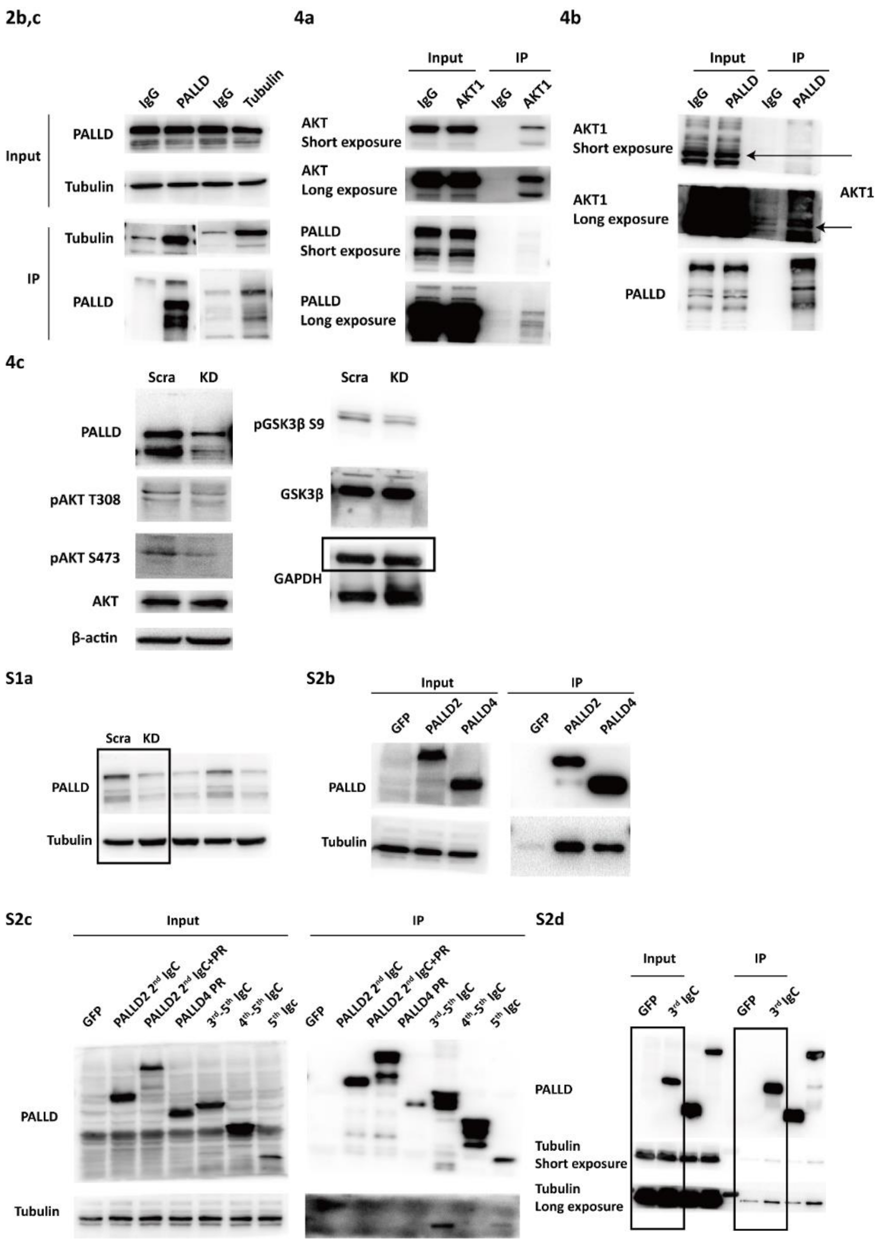

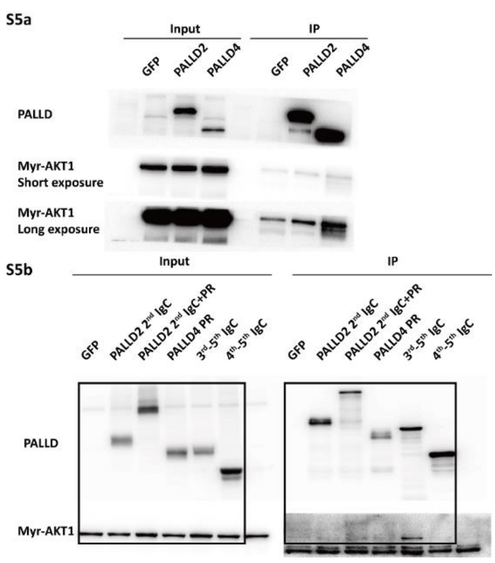

Supplementary Figure 13. Original images of immunoblots.
